# Idiopathic Syringomyelia: A Systematic Scoping Review

**DOI:** 10.3390/jcm15166216

**Published:** 2026-08-11

**Authors:** Renata Martinelli, Luca Massimi

**Affiliations:** 1 Department of Neurosurgery, Fondazione Policlinico Universitario A. Gemelli IRCCS, 00168 Rome, Italy; martinellirenata97@gmail.com; 2 Pediatric Neurosurgery, Fondazione Policlinico Universitario A. Gemelli IRCCS, 00168 Rome, Italy; 3 Faculty of Medicine and Surgery, Università Cattolica del Sacro Cuore, 00168 Rome, Italy

**Keywords:** idiopathic syringomyelia, scoping review, arachnoid web, cerebrospinal fluid dynamics, occult arachnoid pathology, syrinx, arachnoid lysis, pediatric syringomyelia

## Abstract

**Background:** Idiopathic syringomyelia (IS) is defined by exclusion: an intramedullary fluid-filled cavity without Chiari malformation, spinal trauma, tumor, infection, or other identifiable cause. Growing evidence suggests IS is a progressively shrinking category as advanced imaging and intraoperative exploration uncover occult arachnoid or hydrodynamic substrates. To the best of our knowledge, no systematic reviews of the literature have been published on this topic. The authors aim to map operational definitions of IS, summarize proposed pathogenetic mechanisms, characterize diagnostic strategies and their yield, describe management approaches and outcomes, and identify pediatric–adult differences. **Methods:** A scoping review was conducted in accordance with PRISMA-ScR. PubMed and Scopus were searched from inception to May 2026. Eligible sources were original studies of any design addressing IS in pediatric or adult patients, in English. Studies on syringomyelia secondary to Chiari malformation, trauma, tumor, or infection were excluded. Data were extracted across five domains and synthesized narratively. **Results:** Eighteen studies (365 patients; five case reports, nine retrospective cohorts, two morphometric studies, one cine-MRI case–control, one technical note) were included. Operational definitions were markedly heterogeneous. Four pathogenetic mechanisms emerged: subarachnoid CSF obstruction with abnormal intramedullary pulse pressure, occult arachnoid pathology, posterior fossa morphometric variants overlapping with the Chiari spectrum, and persistent central canal as a developmental variant. In the four largest pediatric series (*n* = 214), 91–95% of children remained stable or improved on conservative management at up to 7-year follow-up, with no concordance between syrinx size change and clinical course. In symptomatic adults, targeted arachnoid lysis or web excision yielded clinical improvement in 87% of IS-occult arachnoid web patients; syringo-subarachnoid shunting was occasionally effective but risked neurological deterioration without prior substrate identification. **Conclusions:** The idiopathic label reflects the current limits of diagnostic investigation rather than a fixed nosological entity, since occult arachnoid or hydrodynamic substrates are identified in most adult IS cases when advanced imaging and intraoperative exploration are systematically deployed. Pathogenesis converges on a unified model of subarachnoid CSF obstruction generating abnormal intramedullary pulse pressure. Management should be driven by clinical, not radiological, evolution: arachnolysis or web excision is the primary strategy when an operable substrate is identified, while conservative management remains best supported in clinically stable patients, particularly children.

## 1. Introduction

Syringomyelia is a chronic, progressive disorder characterized by longitudinally oriented fluid-filled cavities within the spinal cord parenchyma or central canal [1,2]. It is best understood as a syndrome rather than a disease, sharing a final common pathway of intramedullary fluid accumulation across distinct pathophysiological mechanisms [1,3]. Contemporary classifications distinguish communicating central-canal cavities, typically associated with hindbrain anomalies and hydrocephalus, non-communicating central canal syringes related to subarachnoid space obstruction, and extracanalicular syringes arising from spinal cord injury, infarction, hemorrhage, or inflammation [4,5].

The best-characterized subtype is syringomyelia associated with Chiari type I malformation, in which pulsatile descent of the cerebellar tonsils impairs cranio-spinal CSF communication and drives fluid into the cord [6]. A second well-defined group, primary spinal syringomyelia (PSS), comprises cavities developing in association with focal or diffuse subarachnoid obstruction of identifiable cause: post-traumatic, post-inflammatory, post-hemorrhagic, or compressive. The mechanism is homologous to that of Chiari-related syringomyelia: subarachnoid block reduces compliance, elevates the pulse pressure, and generates a transmedullary gradient promoting syrinx formation [7,8].

Idiopathic syringomyelia (IS) is a residual, exclusion-based category, operationally defined as an intramedullary cavity without Chiari malformation, hindbrain anomaly, hydrocephalus, trauma, arachnoiditis, tumor, tethered cord, basilar invagination, or other identifiable pathology after appropriate imaging work-up [9]. Several lines of evidence suggest this category is progressively shrinking. First, high-resolution MRI, heavily T2-weighted sequences (CISS/FIESTA), and CT myelography have uncovered subtle arachnoid pathology (webs, pouches, cysts, adhesions) in cases previously labelled idiopathic [9,10,11]. Second, phase-contrast cine-MRI has demonstrated abnormal CSF flow dynamics at the foramen magnum and along the spinal canal in apparently idiopathic patients, supporting occult subarachnoid obstruction as the predominant pathogenetic substrate [12,13]. Third, morphometric studies of the posterior cranial fossa have revealed structural abnormalities that partially overlap with those of Chiari type I and Chiari 0, suggesting a continuum rather than discrete entities [14,15].

Three interpretations have been proposed for IS: a distinct entity related to persistence of the central canal, typically as slit-like cavities [6], an occult form of PSS in which arachnoid pathology escapes routine MRI [10], and a forme fruste of the Chiari spectrum including Chiari 0 and craniocervical compliance abnormalities [14,16]. These interpretations are not mutually exclusive and may apply to different subgroups within the heterogeneous IS population.

Despite this substantial literature, no systematic synthesis of the evidence on IS has been performed to current methodological standards. We aim to fill this gap through a systematic scoping review conducted according to the PRISMA-ScR statement [17]. Specifically, we sought to (i) map how IS has been operationally defined across primary studies; (ii) summarize the proposed pathogenetic mechanisms; (iii) characterize diagnostic strategies and their yield; (iv) describe management approaches and their outcomes; and (v) identify the boundaries between IS, slit-like central canal variants, and occult primary spinal syringomyelia.

## 2. Materials and Methods

This scoping review was designed and is reported in accordance with the PRISMA-ScR statement [17]. A five-stage framework, as demanded for scoping reviews, was adopted: (i) identifying the research question; (ii) identifying relevant studies; (iii) study selection; (iv) charting the data; (v) summarizing and reporting the results [18,19].

### 2.1. Research Questions

The review was structured around the following questions: how is idiopathic syringomyelia defined across primary studies, and what diagnostic criteria are required for its assignment? Which pathogenetic mechanisms have been proposed? What diagnostic work-up has been employed? What management strategies have been applied, and with what clinical and radiological outcomes? Are there clinically relevant differences between pediatric and adult populations?

The Population–Concept–Context (PCC) framework was used to assess eligibility as follows [20]. Population: pediatric and adult patients with a diagnosis of idiopathic syringomyelia. Concept: idiopathic, primary, or unexplained syringomyelia, including studies addressing definition, pathogenesis, imaging, surgical or conservative management, and natural history. Context: any clinical setting with no geographical restriction. Inclusion criteria comprised original studies (case reports, case series, retrospective cohorts, prospective studies, comparative studies, technical notes) addressing IS as defined above. Exclusion criteria comprised studies focused on syringomyelia secondary to identified Chiari malformation, spinal trauma, intramedullary or extradural tumors, spinal infections, or scoliosis-only populations; animal studies; book chapters; non-English full-text; studies for which a full text was not retrievable; and letters to the editor.

### 2.2. Information Sources and Search Strategy

PubMed and Scopus were systematically searched. The following string was applied with field-tag adaptation as needed: (syringomyelia OR syrinx OR hydromyelia OR syringohydromyelia) AND (idiopathic OR primary OR “unknown etiology” OR “unknown etiology” OR noncommunicating OR “non-communicating”). The search covered records from database inception to 5 May 2026.

### 2.3. Study Selection

**Figure 1:**
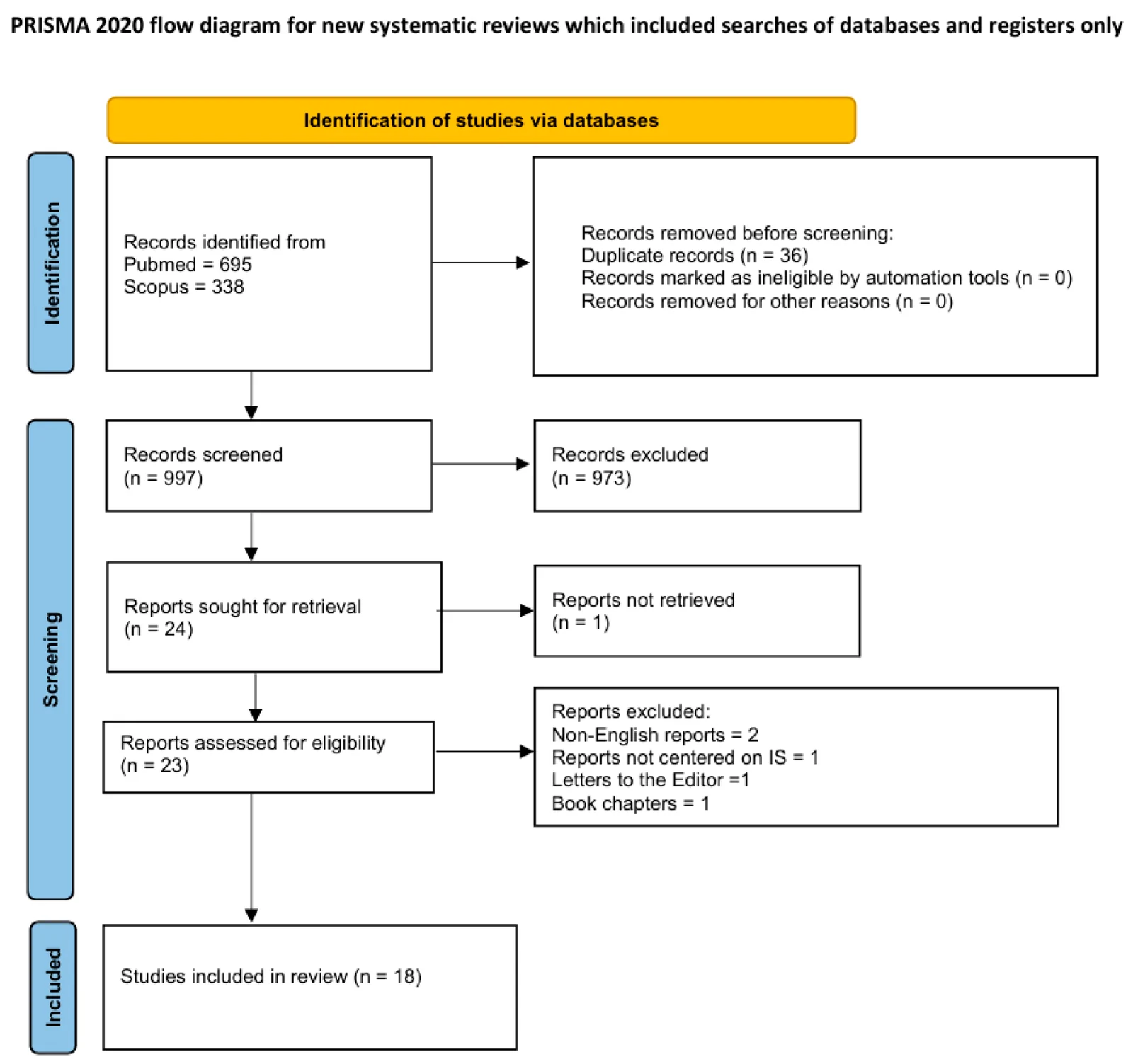
PRISMA flow chart 2020.

Records retrieved from PubMed (*n* = 695) and Scopus (*n* = 338) were imported, and duplicates were removed (*n* = 36), yielding 997 unique records for abstract screening. The authors (R.M. and L.M) independently conducted the abstract screening for eligibility. After screening, 23 records were assessed at full-text level. Five records were excluded (2 non-English; 1 book chapter; 1 not focused on IS; 1 with no retrievable full text and 1 letter to the editor). The final corpus comprised 18 studies. The PRISMA-ScR flow diagram is presented in Figure 1, and the completed PRISMA-ScR checklist is provided in the Supplementary Materials (Supplementary Material File S1). The review protocol was registered on the Open Science Framework (registration DOI: 10.17605/OSF.IO/RPHVZ).

### 2.4. Synthesis Approach

Given the heterogeneity of designs (predominantly retrospective case series and case reports), no quantitative meta-analysis was attempted. Findings were synthesized narratively across the five charting domains, complemented by descriptive tables. Risk of bias was not formally assessed in keeping with PRISMA-ScR methodology, but the methodological strength of each study was qualitatively considered when weighing its contribution.

## 3. Results

### 3.1. Study Selection and Characteristics of Included Studies

Eighteen studies were included in the qualitative synthesis. Designs were heterogeneous: five case reports [21,22,23,24,25], nine retrospective cohort or case series studies [9,10,11,26,27,28,29,30,31], two retrospective comparative morphometric studies [14,15], one case–control phase-contrast cine-MRI study [12], and one technical note with embedded case series [32].

The total number of patients labelled as having idiopathic syringomyelia across the included studies was 365, with substantial heterogeneity in sample size (range 1–98). Gender distribution was variable and study-dependent; both pediatric and adult populations were represented, with pediatric cohorts reported by five studies and adult or mixed populations addressed by the remaining 13 reports. Follow-up duration ranged from cross-sectional to a median of 10 years in the longest series. Detailed study characteristics are reported in Table 1.

### 3.2. Heterogeneity of Operational Definitions

A central finding of this review is that the operational definition of IS is markedly heterogeneous across the literature. Four broad definitional approaches can be distinguished.

A strict definition, requiring exclusion of Chiari malformation, hindbrain anomaly, spinal trauma, tumor, infection, arachnoiditis, tethered cord, basilar invagination, and craniovertebral instability, which requires phase-contrast cine-MRI to rule out occult CSF flow obstruction, is endorsed and applied rigorously in the recent Lu et al. [11] and Sun et al. [31] series. Within this strict framework, IS is essentially a residual category for cases in which no obstructive lesion is identified after exhaustive investigation.

An intermediate definition, applied by Roy et al. [9], Nakamura et al. [27], Davidson et al. [26] and most of the other retrospective series, requires exclusion of the major identifiable causes but does not mandate cine-MRI. Occult arachnoid webs and similar pathologies may therefore be included or excluded depending on intraoperative findings.

**Figure 2:**
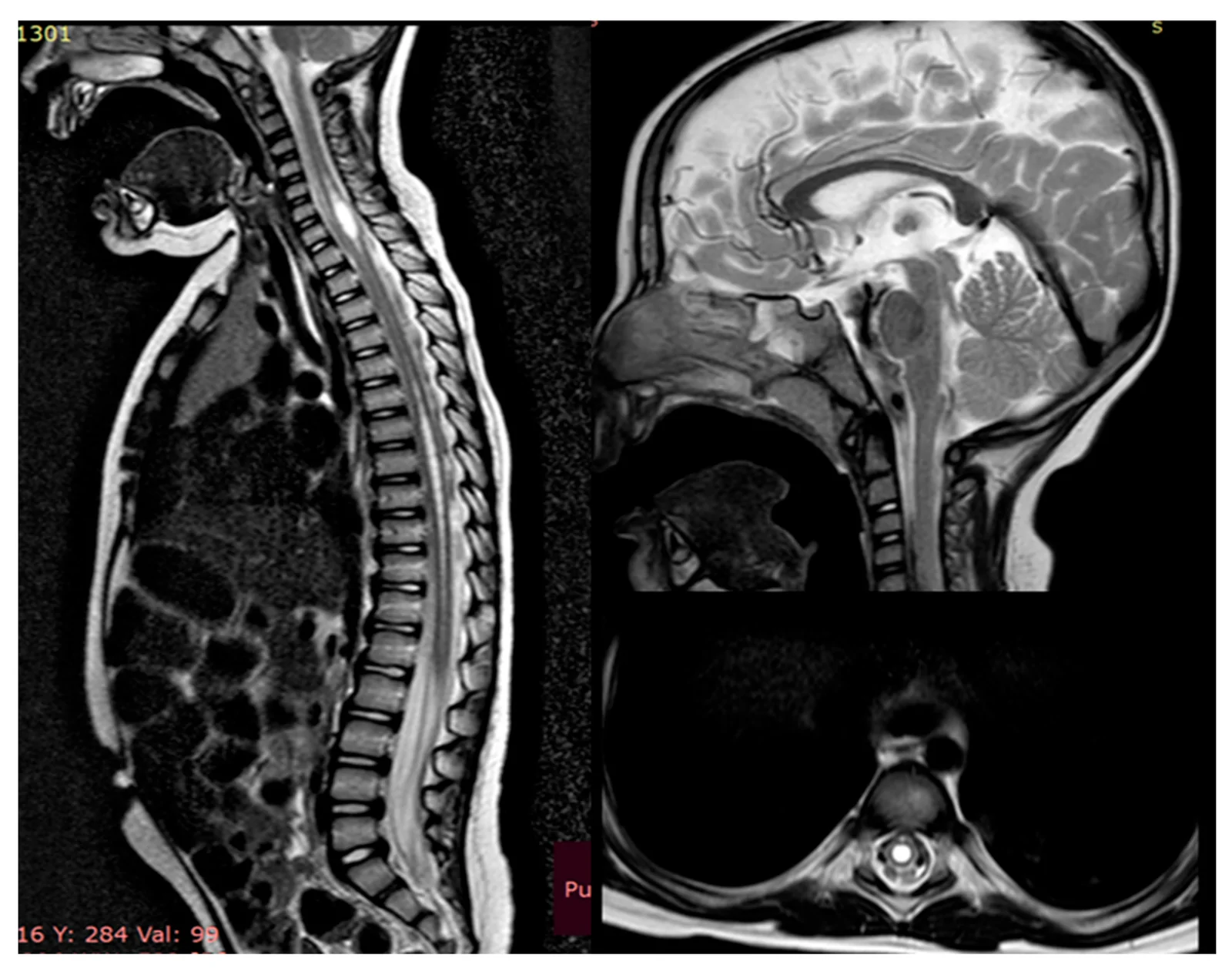
Illustrative example of true idiopathic syringomyelia. A 3-year-old girl with isolated cervical and dorsal syringomyelia. No Chiari malformation, tethered cord, or other spinal cord anomalies are evident.

**Figure 3:**
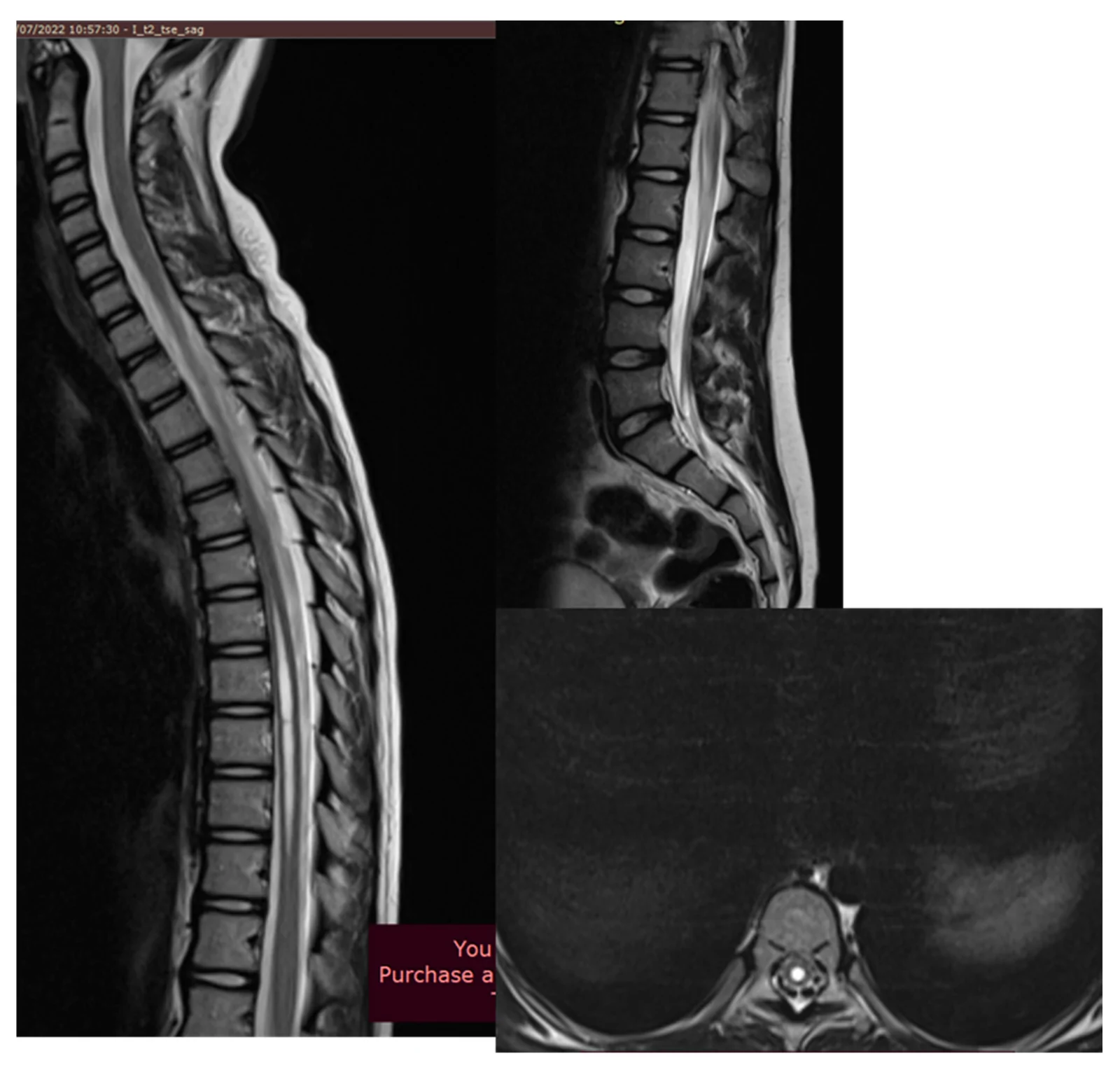
Illustrative example of hydromyelia. Typical isolated dorsal hydromyelia in a 20-year-old girl.

**Figure 4:**
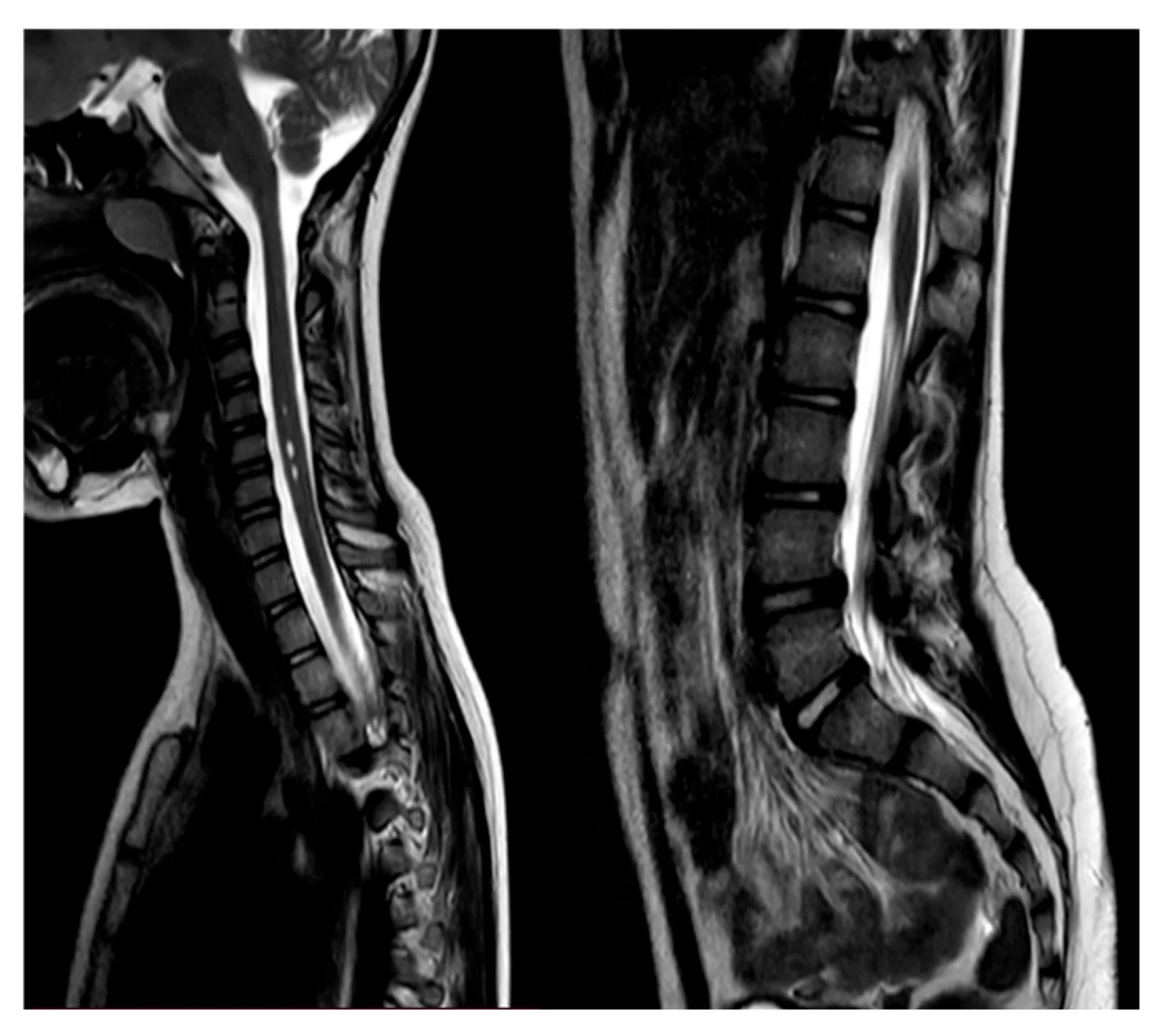
Illustrative example of persistent central canal. Focal dilatation of the cervical central spinal canal as an incidental finding in an 11-year-old girl with scoliosis.

A broad definition, prevalent in pediatric studies [28,29,30], includes any intramedullary cystic cavity ≥0.5–1 mm without an evident cause, often without distinguishing between true syringomyelia and dilatation of the persistent central canal. This point deserves emphasis, since the boundary between true syringomyelia, hydromyelia and persistent central canal is clinically relevant but consistently blurred within this group (Figure 2, Figure 3 and Figure 4). The classical histological distinction defines a true syrinx as an extra-ependymal, glial-lined intramedullary cavity, whereas hydromyelia denotes dilatation of the ependymal-lined central canal, a developmental variant with a reported prevalence of 1.5% in the general population [28]. Crucially, the two entities cannot be reliably distinguished on standard MRI without histological confirmation, as explained by Magge et al., who declared having used a broad definition of syrinx that included dilated central canals [28]. Rodriguez et al. similarly noted that 17 of 98 patients (17.3%) had cavities <2 mm, probably representing normal variants rather than true syringomyelia [30]. This definitional permissiveness might explain the higher prevalence of IS reported in pediatric cohorts.

A contested definition, advanced by Holly and Batzdorf [6] and reiterated in case reports such as Rigsby and Tong [25], questions whether slit-like cavities (narrow, central, symmetrical, often discontinuous, tapering rostrally and caudally) should be considered syringomyelia at all, proposing instead that they represent persistent central canal which consists in a developmental variant rather than a disease. A related reinterpretation was advanced by Nakamura et al., who proposed that idiopathic syringomyelia comprises two distinct entities, a localized form (≤3 vertebral levels) and an “extended” form (≥4 levels), and concluded that the localized type represents congenital enlargement of the central canal of the spinal cord and may be true idiopathic syringomyelia. Rigsby and Tong similarly argued that the slit-like pediatric cavity in their case (0.5–2 mm, spontaneously resolved) was best classified as a persistent central canal rather than a true syrinx [25]. This view supports the broader hypothesis that part of what is labelled “IS”, particularly small, slit-like, localized cavities in pediatric cohorts, may not be pathological at all.

The implications of this definitional heterogeneity are that the “IS” populations described across studies are not directly comparable, and that any aggregate inference about prognosis or management must be interpreted accordingly. The different definitions adopted by the studies included in this review are summarized in Table 2.

### 3.3. Proposed Pathogenetic Mechanisms

Across the 18 included studies, four (non-mutually exclusive) pathogenetic mechanisms emerged.

#### 3.3.1. Subarachnoid Space Obstruction with Abnormal CSF Pulse Pressure

This unifying model articulated by Heiss et al. for PSS [7], who demonstrated that subarachnoid obstruction reduces thecal sac compliance and amplifies pulsatile CSF pressure waves acting on the cord, and conceptually developed in the work of Greitz, who proposed that a relative increase in intramedullary pulse pressure compared to the adjacent subarachnoid space distends the cord through a Venturi-mediated suction effect [1], is supported in the IS context by phase-contrast cine-MRI evidence of altered foramen magnum and spinal CSF flow velocities in IS patients compared with controls [12]. Lu et al. [11] p. 202 explicitly extend this framework by introducing the concept of spinal compliance, analogous to intracranial compliance in hydrocephalus, as the physiological substrate disturbed by occult arachnoid pathology, with restoration of spinal cord pulsation serving as an intraoperative marker of adequate decompression.

#### 3.3.2. Occult Arachnoid Pathology

This is arguably the dominant mechanism in surgically managed adult IS. Mallucci et al., in a series of 10 patients selected from a database of over 800 syringomyelia cases, demonstrated that the great majority harbored occult arachnoid webs, pouches, or cysts identifiable with CT myelography or at surgery [10]. This concept has been amplified by Lu et al., who described 15 patients with idiopathic syringomyelia and occult arachnoid webs (IS-OAW), reporting characteristic radiological signs (the *scalpel sign* and the *girdle sign*), histological evidence of fibrosis with meningothelial cells and psammoma-body calcifications consistent with chronic, non-infectious, non-acute inflammation, and favorable outcomes after targeted arachnolysis at the responsible segment [11]. Davidson et al. provide complementary evidence: in their series of 41 syrinx-to-subarachnoid shunt operations, 15 patients (37%) had IS, and the surgical outcomes did not differ significantly between idiopathic and otherwise classified cases, which might imply a shared obstructive substrate [26].

#### 3.3.3. Posterior Fossa and Craniocervical Morphometric Variants

Two different studies [14,15] have shown that posterior fossa volume, foramen magnum diameter, and cerebellum-to-posterior-fossa ratios in IS deviate from healthy controls and partially overlap with Chiari I, supporting the existence of a continuum of CM-0, CM-1 and IS on an anatomical basis. The clinical relevance of this finding is exemplified by case reports describing successful posterior fossa decompression in highly selected IS patients with morphometric features of “tight cisterna magna” or Chiari 0 [16,23].

#### 3.3.4. Persistent Central Canal as a Developmental Variant

Reconceptualization of slit-like cavities as remnants of the embryonic central canal [6], supported by autopsy data demonstrating incomplete obliteration of the canal into adolescence and young adulthood, provides a developmental, non-pathological mechanism for a substantial subset of small, central, narrow cavities, particularly in pediatric populations [25].

The distribution of pathogenetic mechanisms invoked across the 18 included studies is summarized in Supplementary Material Table S1.

### 3.4. Diagnostic Work-Up

Standard MRI of the cervico-thoracic spine, supplemented by brain imaging to exclude Chiari malformation and hydrocephalus, was universally employed across the included studies. Beyond this baseline, considerable heterogeneity emerged in the use of advanced techniques.

Phase-contrast cine-MRI, regarded as the reference non-invasive technique to identify occult CSF flow obstruction [13], was systematically employed in only a minority of studies: most rigorously by Struck and Haughton in their case–control comparison [12], and selectively by Roy et al. [9] and Nakamura et al. [27]. The recent Lu et al. series privileges high-resolution myelography over cine-MRI for the identification of the responsible segment in IS-OAW, demonstrating subarachnoid block of varying severity from T2 to T10. CT myelography, although invasive, retains a historical and defined role when arachnoid pathology is suspected and not visible on routine MRI [9,10,11]. Heavily T2-weighted high-resolution sequences (CISS, FIESTA) have been advocated as a non-invasive alternative for visualizing arachnoid webs and adhesions and has been proved to be more reliable compared to the invasive conventional myelography [13], although their use across the included studies is uneven.

Intraoperative ultrasonography, employed selectively to confirm the syrinx location and assess arachnoid pathology before durotomy, is described in the surgical series of Davidson et al. [26] and Lu et al. [11]. Intraoperative findings, including direct visualization of thickened, adherent arachnoid, transverse arachnoid septa or focal cord indentation, have repeatedly been shown to upgrade preoperative diagnoses, reclassifying a proportion of “IS” cases as occult PSS [10,11,26].

Routine CSF analysis, brain MRI to exclude hydrocephalus or intracranial mass lesions, and laboratory work-up to exclude infectious or inflammatory etiologies are widely recommended but inconsistently reported across the included studies. The diagnostic yield of extended work-up, defined as the proportion of initially “idiopathic” patients in whom a specific cause was identified by advanced imaging or surgery, varied widely. In Mallucci et al. [10], 9 of 10 patients had arachnoid pathology demonstrated at CT myelography or surgery. In Lu et al. [11], all 15 IS-OAW patients had occult arachnoid pathology confirmed intraoperatively and histologically. In the embedded case series of Roy et al. [9], intraoperative arachnoid abnormalities were identified in a subset of operated patients despite unremarkable preoperative MRI. These findings collectively support the inference that the more thorough the work-up, the smaller the residual idiopathic category becomes. The diagnostic strategies reported across the corpus are summarized in Table 2.

### 3.5. Management Strategies and Outcomes

Across the 18 included studies, management strategies ranged from purely conservative observation in studies dominated by pediatric or radiologically stable populations, to systematic surgical exploration in adult cohorts in which an occult arachnoid substrate was actively sought. The management data are summarized in Supplementary Material Table S2.

#### 3.5.1. Conservative Management

Conservative management, intended as clinical surveillance with serial neurological examination, supplemented by selective imaging follow-up, was the dominant strategy in the pediatric cohorts, in line with previous evidence which proves that children with IS remain asymptomatic, stable, or improve in over 90% of cases and, radiologically, the majority of their syringes (87.5%) remain stable or shrink over time, with no clear correlation between changes in size and changes in symptoms [33]. Magge et al. [28], in the largest multicentric pediatric series with full follow-up data (*n* = 32), reported that 91% of patients remained asymptomatic, stable or improved without surgical intervention; the two operated patients failed to derive clinical benefit. Joseph et al. [29] reached a comparable conclusion in 39 children: all patients were stable or improved, no permanent shunt was placed, and routine surveillance imaging was deemed unnecessary in clinically stable cases. Rodriguez et al. [30], in the largest single-center pediatric series (*n* = 98), confirmed that 49% improved and 28% remained asymptomatically stable without surgery, with only 8% deteriorating. Sun et al. [31], reported that 27 conservatively managed children were asymptomatic, stable or improved at a median radiological follow-up of 6.84 years, and a clinical follow-up of 7.66 years. Kastrup et al. [22] provided the paradigmatic adult counterpart to this evidence, documenting complete spontaneous radiological resolution of a holocord syrinx after 8 years of conservative follow-up, although clinical signs persisted, presumably reflecting irreversible structural cord damage.

Taken together, these data converge on a fundamental concept: in clinically stable patients, particularly in the pediatric population, conservative management is supported by the best evidence currently available, and serial imaging in the absence of clinical change appears to be scarcely relevant [28,29,30,31,33], p. 202.

#### 3.5.2. Surgical Management

Surgical strategies reported across studies reflected the heterogeneity of the underlying pathophysiological assumptions. Three broad approaches emerged.

The first is targeted treatment of an identified arachnoid substrate: laminectomy with excision of arachnoid webs, pouches or cysts, or focal arachnolysis. The use of intraoperative neuromonitoring (IONM) is highly suggested [34] as well as the use of intraoperative ultrasound to identify the ideal place where to start the dura opening under the microscope, in order to avoid direct injury to the spinal cord, which in some cases can be directly adherent to the overlying dura.

Mallucci et al. established this approach as the procedure of choice in adult IS, reporting marked or moderate clinical improvement in 8 of 8 patients who underwent laminectomy plus excision of the obstructive arachnoid lesion, with 6 of 10 syringes collapsing or disappearing radiologically [10]. Clifton et al. provided the earliest demonstration of this principle in a single case in which an occult T3–T6 arachnoid cyst was the operative finding [21], and Lu et al. extended the same principle to the contemporary era, with outcomes comparable to those of a post-traumatic dynamic syringomyelia (PTDS) control group [11]. Sun et al. in their case report performed thoracic adhesiolysis at T11 after pre-operative CISS imaging had identified a suspected adhesion subsequently confirmed intraoperatively [31].

The second approach is cranio-cervical decompression, generally reserved for cases in which a posterior fossa contribution (CM-0 spectrum or abnormal CSF dynamics at the foramen magnum) is hypothesized. In these cases, careful intraoperative assessment should be directed toward identifying potential causes of residual or recurrent CSF obstruction at the level of the foramen of Magendie, including tonsillar herniation overlying the outlet, compression by vermian branches of the posterior inferior cerebellar artery (PICA), arachnoid veils partially or completely occluding the foramen, and postoperative pseudomeningocele formation with associated arachnoid adhesions [35,36]. In this review, only Chen et al. reported clinical and radiological improvement after suboccipital plus cervical decompression in an acute-onset case in which intraoperative dural vascular engorgement was identified [23].

**Figure 5:**
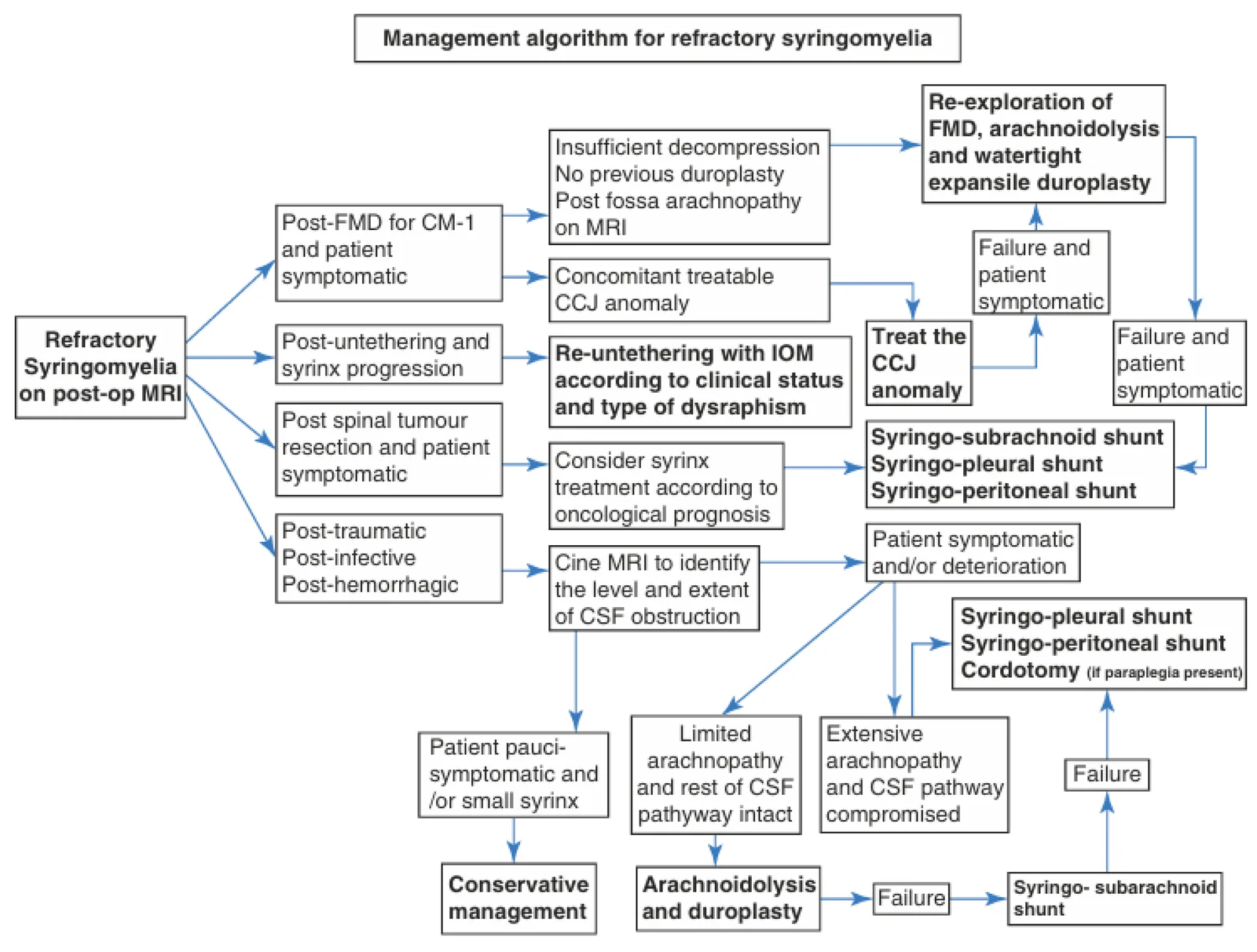
Proposed decision-making workflow for the management of idiopathic syringomyelia. Original figure by Gallo et al. [2].

The third approach is syringo-subarachnoid (SS) shunting. Davidson et al. [26] reported the largest contemporary surgical series of SS shunting in syringomyelia (*n* = 41, of whom 15 were IS), with 90% radiological reduction, 98% clinical stabilization or improvement and a 7% reoperation rate; the outcomes did not differ significantly between IS and other etiologies. Lin et al. [24] and Soo et al. [32] reported similarly favorable outcomes with conventional and silastic-wedge SS shunts, respectively. However, Mallucci et al. had already shown that isolated syringo-shunting performed without prior identification and treatment of the arachnoid substrate carries a risk of deterioration (2 of 2 patients shunted without exploration deteriorated, one becoming paraplegic) [10]. Shunting should be therefore reserved for situations in which no operable substrate can be identified or in which decompression and arachnolysis have failed [9,37]. Gallo et al. [2] proposed an evidence-based decision-making workflow integrating these management strategies, presented in Figure 5. A fourth, less invasive option emerging from the corpus is therapeutic lumbar puncture: Joseph et al. [29] reported significant pain reduction in 4 of 4 pediatric patients with progressive back pain, suggesting a possible role in selected symptomatic cases where formal CSF dynamics studies could also be relevant to the diagnosis.

### 3.6. Pediatric Versus Adult Populations

Five of the 18 included studies focused exclusively on pediatric cohorts, accounting for 214 of the 365 IS patients in the review (58.4%) [25,28,29,30,31]. The remaining studies addressed adult or mixed populations. The contrast between the two age strata is informative. In the pediatric studies, the predominant pattern is one of benign natural history with a low rate of operable substrates: across the four largest pediatric series with full clinical follow-up [28,29,30,31], 168 of 177 children with available clinical follow-up (94.9%) were asymptomatic, stable or improved on conservative management, and only 4 (1.9%) of 213 patients in total (not counting the pediatric case report by Rigbsy et al. [25]) underwent a surgical procedure. When performed, surgery often failed to alter the clinical course: in Magge et al., neither operated patient derived clinical benefit [28], and in Sun et al., the single operated child showed radiological improvement but no clinical change [31]; in Rodriguez et al., the single shunted patient deteriorated, although the deterioration was largely attributable to the underlying muscular dystrophy [30]. Across all four series, the radiological behavior of the syrinx did not predict clinical outcome. Notably, Joseph et al. [29] additionally reported therapeutic lumbar punctures in 4 of 39 children (10.3%) with progressive back pain, all with significant pain reduction at follow-up, which were counted separately from surgical procedures.

In the adult studies, the pattern is markedly different. When actively searched for, an occult arachnoid substrate is frequently identified in adult IS cases. Mallucci et al. demonstrated, on systematic addition of myelography and intraoperative exploration, that 9 of 10 adult patients (90%) initially labelled as idiopathic harbored an occult arachnoid web, pouch or cyst [10]. Lu et al., in the contemporary IS-OAW cohort, prospectively investigated 15 patients with mandatory pre-operative myelography, intraoperative ultrasound and histopathological confirmation, and identified an occult arachnoid web at the responsible spinal segment in 15 of 15 cases [11]. Arachnolysis at the identified segment produced significant clinical improvement in 87% of IS-OAW patients, compared with 44% in the post-traumatic syringomyelia control group (*p* = 0.011). These findings progressively narrow the residual “true idiopathic” category [7,37]. Davidson et al. [26] added the complementary surgical lesson on the management end: in their consecutive series of 41 patients treated with syringo-subarachnoid shunting, the 15 patients with idiopathic syringomyelia achieved a favorable outcome (improved or stable) in all cases, with no significant difference from patients with a well-defined etiology, confirming that once an occult substrate is either identified and addressed, the IS label does not predict surgical outcome.

Two observations can be made regarding the pediatric and adult cohorts. First, the discordance between radiological and clinical evolution is a recurring phenomenon at both age extremes, and it has direct implications for the design of surveillance protocols and surgical decision-making. Second, the “shrinking-idiopathic” phenomenon documented in the adult cohorts has now been observed, even if rarely (1/28, 3.6%), within the strictest pediatric cohort reviewed [11]. These findings point toward a shared hydrodynamic substrate underlying both pediatric and adult IS, suggesting that the label “idiopathic” reflects the current limits of diagnostic investigation rather than a stable nosological category.

## 4. Discussion

This scoping review synthesizes 18 studies and 365 patients across nearly four decades of the literature. Beyond aggregating the available evidence, four conceptual contributions emerge from the synthesis. First, the IS category has contracted progressively as diagnostic work-up has extended, from 90% diagnostic yield with the addition of myelography [10] to 100% intraoperative confirmation of occult arachnoid webs with contemporary combined imaging and histology [11]; even in the most rigorously selected pediatric cohort published to date, one in twenty-eight patients was reclassified at surgery [31]. Second, the reviewed articles consolidate the three historical models of pathophysiology of syringes under a unified pathogenetic framework centered on subarachnoid CSF obstruction, Venturi-mediated transmedullary pressure gradient and perivascular fluid transmission [1,2,7], with direct therapeutic implications. Third, the heterogeneity of inclusion criteria across studies introduces a categorical ambiguity between true syringomyelia, hydromyelia and persistent central canal that has never been formally resolved in the literature and that affects any inferential conclusion. Fourth, the absence of correlation between syrinx size change and clinical course, documented at both age extremes [10,26,28,30,31], is consistent enough across cohorts to warrant elevation from an incidental finding to a decision-making principle: clinical, not radiological, evolution should drive surveillance and surgical decisions. Each of these contributions is elaborated in the paragraphs that follow.

The body of literature examined in this review points consistently in one direction: what is currently labelled idiopathic syringomyelia is a shrinking category, contracting with each advance in imaging resolution, intraoperative technique, and pathophysiological reasoning. Mallucci et al. showed that, when myelography was added to standard imaging, 9 of 10 apparently idiopathic adult cases harbored an occult arachnoid lesion [10]. Two decades later, Lu et al. confirmed and extended this observation in the contemporary era, with all 15 IS-OAW cases verified intraoperatively and histologically [11]. Between these two endpoints, Struck and Haughton demonstrated abnormal CSF dynamics at the foramen magnum in 5 of 8 IS patients using phase-contrast cine-MRI, again pointing to an unrecognized CSF-flow substrate rather than a true absence of cause [12]. Most recently, Sun et al., despite adopting one of the strictest operational definitions of pediatric IS in the literature, still reported one patient whose syringomyelia was ultimately reclassified intraoperatively as an arachnoid adhesion [31] p. 20. This single case, included in an otherwise rigorously selected cohort, represents the pivotal demonstration that the IS category contracts as the diagnostic work-up improves, and is not a fixed nosological entity. Much of what is currently labelled idiopathic might be, more accurately, occult primary spinal syringomyelia in the sense proposed by Heiss et al. [7], with the “idiopathic” label marking the boundary of current diagnostic capability rather than a true absence of cause.

This progressive narrowing of the idiopathic category is therefore inseparable from the evolution of imaging tools. From the studies evaluated in this review, a clear hierarchy of investigative tools emerges, in which each step further constrains the residual idiopathic category. Standard structural MRI remains the level at which most patients are first labelled as IS, but is now widely recognized as insufficient for differential diagnosis [2,13,37]. Phase-contrast cine-MRI added quantitative information on CSF flow at the foramen magnum and, when applied at the spinal level, identified subarachnoid blocks in a substantial proportion of otherwise idiopathic cases [12,13]. High-resolution, heavily T2-weighted sequences, such as FIESTA, CISS or balanced steady-state free precession, have more recently become the most powerful non-invasive tool for visualizing thin arachnoid webs, septations and focal adhesions, as demonstrated by Sun et al. [31] and Lu et al. [11]. CT myelography retains a role in the few cases where MRI sequences remain inconclusive [27], and intraoperative ultrasound provides final confirmation in selected series [26]. The practical implication of this hierarchy is that the diagnosis of IS should be considered provisional unless cine-MRI and CISS/FIESTA imaging have both been performed and reported as unremarkable.

A further consistent thread across studies is the discordance between radiological and clinical outcomes, which has direct implications for how these patients are followed. Rodriguez et al. [30] explicitly reported no concordance between syrinx size change and clinical course in their 98 pediatric patients, and Magge et al. [28] reached the same conclusion in their multicentric cohort. Sun et al. [31] confirmed this on long-term follow-up: over half of syringes improved radiologically, yet these changes did not associate with clinical course. Even in adult surgical series, the same discordance recurs [10,22,26]. The implication for clinical practice is unambiguous: both surgical and follow-up decisions in IS should be driven primarily by clinical evolution, not by serial radiological measurements.

Three classical models have historically dominated the understanding of syrinx formation. Gardner’s hydrodynamic theory proposed that CSF from the fourth ventricle enters the spinal cord through a patent central canal driven by a water-hammer pulsatile force [38]. Williams refined this with a cranio-spinal pressure dissociation model, in which Valsalva-induced venous congestion generates a rostral to caudal CSF pressure gradient across a foraminal obstruction, propelling fluid into the cord through a ball-valve mechanism. Both models, however, depend on a direct communication between the subarachnoid space and the cavity, an anatomical prerequisite subsequently disproved by cadaveric evidence that most syringes are extracanalicular and do not communicate with the fourth ventricle [3]. The intramedullary pulse pressure model advanced by Greitz [1] resolves this paradox and forms the basis of the contemporary framework: pulsatile CSF flow through a narrowed subarachnoid space generates a Venturi-mediated suction effect, whereby the accelerating CSF column produces a transmedullary pressure gradient (Bernoulli’s principle) that draws interstitial fluid outward through the Virchow–Robin perivascular spaces and, over time, into the developing intramedullary cavity. This mechanism identifies the subarachnoid obstruction, rather than the cavity itself, as the primary pathological event, and has been subsequently integrated by Klekamp [37] and Heiss et al. [7] with the empirical observation that even minor subarachnoid obstructions, including occult arachnoid webs, focal adhesions and posterior fossa morphometric variants, can generate hydrodynamic conditions sufficient to drive syrinx formation. The studies included in this review converge with remarkable consistency on this integrating framework. Mallucci et al. provided the founding clinical demonstration of this model in the adult IS population [10], Struck and Haughton quantified its hemodynamic correlate at the foramen magnum [12], and Lu et al. supplied contemporary surgical and histopathological confirmation [11]. Two further studies extended this framework to cranial predisposing factors. Bogdanov et al. [14] and Özkal et al. [15] showed that IS patients share, at least in part, the reduced posterior-fossa volume and altered cerebellum-to-posterior-fossa ratios characteristic of Chiari I malformation, supporting a continuum between CM-1, CM-0 and IS at the cranial level. This anatomical observation provides a plausible explanation for why some patients develop cervical subarachnoid blocks even in the absence of frank tonsillar herniation, and locates the so-called Chiari-zero hypothesis along the same pathophysiological spectrum centered on subarachnoid CSF obstruction. The practical consequences are that diagnostically, caution is warranted before labelling any patient as idiopathic without careful posterior-fossa imaging including morphometric measurement and quantitative cine-MRI, and therapeutically, if the substrate is a focal subarachnoid obstruction, then arachnolysis or web excision is the rational primary treatment rather than shunting the cavity itself.

These pathophysiological considerations translate directly into the management picture that emerges from the reviewed studies. In stable, oligosymptomatic patients, particularly in the pediatric population, conservative management with selective clinical and radiological follow-up is supported by the largest cohorts and by the longest available follow-up data. In symptomatic adult patients in whom advanced imaging or intraoperative exploration identifies an arachnoid substrate, targeted laminectomy with arachnolysis or web excision yields the most favorable outcomes. Cranio-cervical decompression is reserved for cases with a documented or strongly suspected posterior-fossa contribution, with careful intraoperative assessment directed toward occult causes of residual CSF obstruction at the foramen of Magendie. Syringo-subarachnoid shunting, although effective in selected series [24,26,32], should be reserved as a salvage option when no operable substrate is identifiable or when arachnolysis has failed, as taught by the early lesson of Mallucci et al. on the risk of neurological deterioration after isolated shunting without prior substrate identification [10]. Therapeutic lumbar puncture, as proposed by Joseph et al. [29], may have a role in selected symptomatic pediatric cases, though prospective evaluation remains lacking.

Three features distinguish the pediatric IS literature and deserve separate consideration. First, the natural history is overwhelmingly benign: across the four largest pediatric series, the great majority of children were stable or improved on conservative management, including on long-term follow-up of approximately 7 years. Second, a proportion of pediatric “syringes” may represent persistent central canal variants, developmental rather than pathological findings, raising a real risk of overdiagnosis when small slit-like cavities are interpreted as true syringes. Third, the discordance between radiological and clinical evolution is more pronounced in children than in adults, reinforcing a clinically driven rather than radiologically driven follow-up strategy. Taken together, these three features justify a pediatric management approach focused on clinical surveillance, with selective imaging and a high threshold for surgical intervention.

Across both populations, the data converge on the hypothesis that what is labelled IS today is likely a heterogeneous group in which occult subarachnoid pathology, such as arachnoid webs, focal adhesions, subtle CSF flow obstruction, or reduced posterior-fossa compliance, remains undetected. As imaging resolution improves and intraoperative exploration becomes more systematic, the residual, truly idiopathic category will continue to shrink. This diagnosis should therefore be understood as provisional, subject to revision with each advance in diagnostic capability, and should prompt rather than foreclose further investigation.

## 5. Limitations and Future Perspectives

The present review spans nearly four decades and includes several methodologically rigorous contributions, such as quantitative cine-MRI, mandatory pre-operative myelography with histological confirmation, long-term follow-up with strict exclusion criteria, and cranial morphometric comparison with controls. The limitations, however, remain substantial: 13 of 18 studies are retrospective, 5 are case reports, sample sizes are modest, follow-up protocols are heterogeneous, and prospective designs with standardized cine-MRI and CISS are essentially absent. This significant heterogeneity among the studies precludes a systematic review. Formal risk-of-bias appraisal was not performed, in keeping with PRISMA-ScR methodology, and the total of 365 patients should not be read as an epidemiological estimate, given possible cohort overlap.

These limitations identify three priorities for future research: a shared operational definition of IS that requires the negative result of an extended work-up before the idiopathic label is applied, prospective multicentric registries of patients meeting this definition to estimate the size and natural history of the residual “true IS” category, and a dedicated investigation of the clinico-radiological discordance documented across pediatric and adult cohorts, ideally combining cine-MRI biomarkers with patient-reported outcomes.

## 6. Conclusions

This scoping review of 18 studies including 365 patients delineates a diagnostic category whose boundaries have been contracting steadily. Five conclusions can be drawn with reasonable confidence from the available evidence.

First, the idiopathic label denotes the current limit of the diagnostic work-up rather than a fixed nosological entity: when advanced imaging and intraoperative exploration are systematically deployed, an occult arachnoid or hydrodynamic substrate is identified in the great majority of adult IS cases [10,11,12].

Second, the pathogenesis of IS converges on a single unifying framework of focal or diffuse subarachnoid CSF obstruction generating abnormal intramedullary pulse pressure through a Venturi-mediated transmedullary gradient [1,2,7], a model with direct therapeutic implications.

Third, management decisions should be driven by clinical, not radiological, evolution: clinico-radiological discordance recurs consistently across pediatric and adult cohorts [10,26,28,30,31].

Fourth, when an operable arachnoid substrate is identified, arachnolysis or web excision is the primary surgical strategy, while syringo-subarachnoid shunting should be reserved for cases in which no substrate is demonstrable or arachnolysis has failed, given the documented risk of neurological deterioration when shunting is performed without prior substrate identification [10].

Fifth, conservative management is best supported for clinically stable patients, particularly in the pediatric population, where the natural history is overwhelmingly benign: 168 of 177 children with clinical follow-up (94.9%) were asymptomatic, stable or improved across the four largest pediatric series, with only 4 of 213 (1.9%) undergoing surgery [28,29,30,31]. Future research should adopt a shared operational definition of IS based on a negative extended work-up, including phase-contrast cine-MRI and heavily T2-weighted sequences, and should explicitly address the boundary between true syringomyelia, hydromyelia, and persistent central canal. Without this, the “idiopathic” label reflects only the current limits of investigation.

## Figures and Tables

**Table 1:** Synthesis of included studies.

Author, Year	Study Design	N (IS)	Population	Key Finding
Clifton et al., 1987 [21]	Case report	1	Adult	Occult intradural arachnoid cyst; decompression resolved the syrinx.
Mallucci et al., 1997 [10]	Retrospective series	10	Adult	9 of 10 had occult arachnoid webs, pouches or cysts demonstrated on myelography or at surgery.
Kastrup et al., 2001 [22]	Case report	1	Adult	Complete spontaneous radiological resolution of IS at 8-year follow-up.
Bogdanov et al., 2004 [14]	Case-control (morphometric)	17	Adult	IS shares posterior fossa under-development with CM-1; 5/17 with spontaneous syrinx drainage.
Chen et al., 2004 [23]	Case report	1	Adult	Acute IS attributed to dural vascular engorgement; suboccipital + cervical decompression led to clinical and radiological improvement.
Lin et al., 2006 [24]	Case report	1	Adult	SSS produced rapid clinical improvement in thoracic IS.
Nakamura et al., 2009 [27]	Retrospective series	15	Adult	Two morphological entities identified: localized IS (*n* = 12, benign, conservative) and extended IS (*n* = 3, progressive, surgical).
Struck & Haughton, 2009 [12]	Case–control (cine-MRI)	8	Adult	Peak systolic caudal CSF velocity significantly higher in IS than in controls; synchronous bidirectional flow and jets in 5/8 IS, 0/6 controls.
Magge et al., 2011 [28]	Retrospective multicentric	48 (32 with FU)	Pediatric	91% asymptomatic/stable/improved on conservative management; no concordance between syrinx size and clinical course; surgery (*n* = 2) provided no benefit.
Roy et al., 2011 [9]	Retrospective + narrative review	8	Adult	Convergence on SAS-obstruction/intramedullary pulse-pressure model; conservative management in 6/8; SS shunting as last resort.
Joseph et al., 2013 [29]	Retrospective series	39	Pediatric	Pediatric IS is benign, without necessary radiological FUP; therapeutic LP achieved significant pain reduction in 4/4 patients.
Soo et al., 2014 [32]	Technical note + case series	5	Adult	Silastic-wedge SS shunt: 5/5 clinically improved and radiologically reduced/stable.
Rodriguez et al., 2015 [30]	Retrospective series	98	Pediatric	49% improved, 28% asymptomatic stable, 8% deteriorated; no concordance between radiological and clinical course.
Davidson et al., 2017 [26]	Retrospective surgical series	15 (of 41)	Adult	Whole cohort: 90% radiological reduction, 98% clinical stabilization/improvement, 7% reoperation.
Rigsby et al., 2021 [25]	Case report	1	Pediatric	Reinterpretation of small pediatric “IS” as persistent central canal; spontaneous resolution at 3-year follow-up.
Lu et al., 2025 [11]	Retrospective comparative	15	Adult	100% diagnostic yield of mandatory CT myelography + intraoperative ultrasound + histology: all 15 with confirmed occult arachnoid webs (IS-OAW).
Özkal et al., 2025 [15]	Case–control (morphometric)	54	Adult	Reduced posterior-fossa area and altered cerebellum/PF ratio in IS support a continuum CM-1/CM-0/IS at the cranial level.
Sun et al., 2025 [31]	Retrospective series	28	Pediatric	53.6% radiological improvement over median 6.84-year FU; 1/28 (3.6%) reclassified at surgery as occult T11 arachnoid adhesion, illustrating “shrinking-idiopathic” phenomenon.

Abbreviations: CM-0, Chiari malformation type 0; CM-1, Chiari malformation type 1; CSF, cerebrospinal fluid; FU, follow-up; IS, idiopathic syringomyelia; IS-OAW, idiopathic syringomyelia with occult arachnoid webs; LP, lumbar puncture; PF, posterior fossa; PSS, primary spinal syringomyelia; SM, syringomyelia; SS shunt, syringo-subarachnoid shunt.

**Table 2:** Operational definitions of idiopathic syringomyelia and diagnostic work-up.

Author, Year	Definition Adopted	Exclusion Criteria Specified	Cine-MRI	Lab Work-Up
Clifton et al., 1987 [21]	Syringomyelia without identifiable cause on standard work-up of the era	Implicit (pre-MRI era; myelographic)	No (myelography)	No
Mallucci et al., 1997 [10]	Symptomatic syringomyelia without recognizable predisposing factors	Implicit (standard MRI)	No (pre-routine; myelography)	No
Kastrup et al., 2001 [22]	Holocord IS without CM, tumor, trauma, or arachnopathy on MRI	Yes (clinical + MRI)	No	No
Bogdanov et al., 2004 [14]	IS = SM without CM and without identifiable cause	Yes (rigorous; no CM, no tumor, no trauma)	No	No
Chen et al., 2004 [23]	Acute IS without CM, tumor, trauma, infection	Yes (clinical + MRI)	No	Yes (B12, syphilis serology)
Lin et al., 2006 [24]	IS = syringomyelia without identifiable cause on MRI	Implicit	No	No
Nakamura et al., 2009 [27]	Syringomyelia without CM, tumor, trauma, post-laminectomy or arachnoiditis	Yes	Yes	No
Struck et al., 2009 [12]	Syringomyelia without CM, tumor, substantial trauma	Yes	Yes	No
Magge et al., 2011 [28]	Pediatric syringomyelia without CM, tumor, trauma, congenital cord anomalies, scoliosis	Yes	No	No
Roy et al., 2011 [9]	IS = no CM-1, no tumor, no trauma, no post-laminectomy	Yes	Selectively (in symptomatic operated cases)	No
Joseph et al., 2013 [29]	IS = syringomyelia without CM-1, tumor, trauma or other identifiable cause	Yes	No (myelography)	Yes (LP opening pressure in selected cases)
Soo et al., 2014 [32]	IS = syringomyelia not associated with CM, malignancy, tethering, trauma, arachnoiditis	Implicit	NA	No
Rodriguez et al., 2015 [30]	Syringohydromyelia ≥ 0.5 mm × ≥ 1 vertebral level, exclusion of known causes	Yes	No	No
Davidson et al., 2017 [26]	IS = syringomyelia without identifiable cause on MRI;	Yes	No	No
Rigsby et al., 2021 [25]	Distinction between slit-like syrinx (persistent central canal) and true syringomyelia	Yes	No	No
Lu et al., 2025 [11]	Apparently idiopathic syringomyelia at extended work-up is revealed to harbor occult arachnoid webs (IS-OAW);	Yes	Selectively (CT myelography)	Yes (CSF, blood, urine; histology + IHC)
Özkal et al., 2025 [15]	Syringomyelia without identifiable cause + active exclusion of CM-like symptoms (to exclude CM-0)	Yes	No	No
Sun et al., 2025 [31]	Pediatric syringomyelia without primary identifiable pathology, also excluding CCJ/CM-like features	Yes	Selectively (pre-op CISS)	Yes (LP performed in 1 case)

CCJ, cranio-cervical junction; CISS, constructive interference in steady state; CM, Chiari malformation; LP, lumbar puncture; PTDS, post-traumatic/post-arachnoiditis dynamic syringomyelia; SM, syringomyelia.

## Data Availability

No new data were created or analyzed in this study. Data sharing is not applicable to this article.

## Supplementary Materials

The following supporting information can be downloaded at: https://www.mdpi.com/article/10.3390/jcm15166216/s1.

## Abbreviations

The following abbreviations are used in this manuscript:

CCJ	Cranio-cervical junction
CISS	Constructive interference in steady state
CM-0	Chiari malformation type 0
CM-1/CM-I	Chiari malformation type 1
CSF	Cerebrospinal fluid
CT	Computed tomography
FIESTA	Fast imaging employing steady-state acquisition
IONM	Intraoperative neuromonitoring
IS	Idiopathic syringomyelia
IS-OAW	Idiopathic syringomyelia with occult arachnoid web
MRI	Magnetic resonance imaging
PCC	Population–Concept–Context
PF	Posterior fossa
PICA	Posterior inferior cerebellar artery
PRISMA-ScR	Preferred Reporting Items for Systematic reviews and Meta-Analyses extension for Scoping Reviews
PSS	Primary spinal syringomyelia
PTDS	Post-traumatic dynamic syringomyelia
SS	Subarachnoid space
SS	Syringo-subarachnoid

## References

[1] Greitz D. (2006). Unraveling the riddle of syringomyelia. Neurosurg. Rev..

[2] Gallo P., Kaliaperumal C., Di Rocco C. (2022). The Management of Idiopathic and Refractory Syringomyelia. Advances and Technical Standards in Neurosurgery.

[3] Milhorat T.H., Capocelli A.L., Anzil A.P., Kotzen R.M., Milhorat R.H. (1995). Pathological basis of spinal cord cavitation in syringomyelia: Analysis of 105 autopsy cases. J. Neurosurg..

[4] Batzdorf U. (2005). Primary spinal syringomyelia: Invited submission from the Joint Section Meeting on Disorders of the Spine and Peripheral Nerves, March 2005. J. Neurosurg. Spine.

[5] Milhorat T.H. (2000). Classification of syringomyelia. Neurosurg. Focus.

[6] Holly L.T., Batzdorf U. (2002). Slitlike syrinx cavities: A persistent central canal. J. Neurosurg. Spine.

[7] Heiss J.D., Snyder K., Peterson M.M., Patronas N.J., Butman J.A., Smith R.K., DeVroom H.L., Sansur C.A., Eskioglu E., Kammerer W.A. (2012). Pathophysiology of primary spinal syringomyelia: Clinical article. J. Neurosurg. Spine.

[8] Oldfield E.H., Muraszko K., Shawker T.H., Patronas N.J. (1994). Pathophysiology of syringomyelia associated with Chiari I malformation of the cerebellar tonsils: Implications for diagnosis and treatment. J. Neurosurg..

[9] Roy A.K., Slimack N.P., Ganju A. (2011). Idiopathic syringomyelia: Retrospective case series, comprehensive review, and update on management. Neurosurg. Focus.

[10] Mallucci L., Stacey R.J., Miles J.B. (1997). Idiopathic syringomyelia and the importance of occult arachnoid webs, pouches and cysts. Br. J. Neurosurg..

[11] Lu C., Yin M., Yuan F., Ding C., Wang X., Jian F. (2025). A Novel Clinical Insight Into Idiopathic Syringomyelia with Occult Arachnoid Webs: Neuropathological Features, Differential Diagnosis, and Surgical Strategy. Neurospine.

[12] Struck A.F., Haughton V.M. (2009). Idiopathic Syringomyelia: Phase-Contrast MR of Cerebrospinal Fluid Flow Dynamics at Level of Foramen Magnum. Radiology.

[13] Mauer U.M., Freude G., Danz B., Kunz U. (2008). Cardiac-gated phase-contrast magnetic resonance imaging of cerebrospinal fluid flow in the diagnosis of idiopathic syringomyelia. Neurosurgery.

[14] Bogdanov E.I., Heiss J.D., Mendelevich E.G., Mikhaylov I.M., Haass A. (2004). Clinical and neuroimaging features of “idiopathic” syringomyelia. Neurology.

[15] Özkal B., Özçelik H. (2025). Idiopathic Syringomyelia: Diagnostic Value of Cranial Morphometric Parameters. Brain Sci..

[16] Tubbs R.S., Elton S., Grabb P., Dockery S.E., Bartolucci A.A., Oakes W.J. (2001). Analysis of the Posterior Fossa in Children with the Chiari 0 Malformation. Neurosurgery.

[17] Tricco A.C., Lillie E., Zarin W., O’Brien K.K., Colquhoun H., Levac D., Moher D., Peters M.D.J., Horsley T., Weeks L. (2018). PRISMA Extension for Scoping Reviews (PRISMA-ScR): Checklist and Explanation. Ann. Intern. Med..

[18] Levac D., Colquhoun H., O’Brien K.K. (2010). Scoping studies: Advancing the methodology. Implement. Sci..

[19] Arksey H., O’Malley L. (2005). Scoping studies: Towards a methodological framework. Int. J. Soc. Res. Methodol..

[20] Peters M.D., Godfrey C., McInerney P., Khalil H., Larsen P., Marnie C., Pollock D., Tricco A.C., Munn Z. (2022). Best practice guidance and reporting items for the development of scoping review protocols. JBI Evid. Synth..

[21] Clifton A.G., Ginsberg L., Webb W.J.S., Valentine A.R. (1987). Idiopathic spinal arachnoid cyst and syringomyelia. Br. J. Radiol..

[22] Kastrup A., Nägele T., Topka H. (2001). Spontaneous resolution of idiopathic syringomyelia. Neurology.

[23] Chen J., Chen C., Lee C., Huang M., Chen T., Weng M. (2004). Acute Idiopathic Syringomyelia: A Case Report. Kaohsiung J. Med. Sci..

[24] Lin J.W., Lin M.-S., Lin C.M., Tseng C.H., Tsai S.H., Kan I.H., Chiu W.T., Chang J.W., Katayama Y., Yamamoto T. (2006). Idiopathic syringomyelia: Case report and review of the literature. Advances in Functional and Reparative Neurosurgery.

[25] Rigsby R.K., Tong K.A. (2021). Spontaneous Resolution of Diffuse Idiopathic Slit-Like “Syrinx” in a Pediatric Patient. Cureus.

[26] Davidson K.A., Rogers J.M., Stoodley M.A. (2018). Syrinx to Subarachnoid Shunting for Syringomyelia. World Neurosurg..

[27] Nakamura M., Ishii K., Watanabe K., Tsuji T., Matsumoto M., Toyama Y., Chiba K. (2009). Clinical Significance and Prognosis of Idiopathic Syringomyelia. J. Spinal Disord. Tech..

[28] Magge S.N., Smyth M.D., Governale L.S., Goumnerova L., Madsen J., Munro B., Nalbach S.V., Proctor M.R., Scott R.M., Smith E.R. (2011). Idiopathic syrinx in the pediatric population: A combined center experience: Clinical article. J. Neurosurg. Pediatr..

[29] Joseph R.N., Batty R., Raghavan A., Sinha S., Griffiths P.D., Connolly D.J.A. (2013). Management of isolated syringomyelia in the paediatric population—A review of imaging and follow-up in a single centre. Br. J. Neurosurg..

[30] Rodriguez A., Kuhn E.N., Somasundaram A., Couture D.E. (2015). Management of idiopathic pediatric syringohydromyelia. J. Neurosurg. Pediatr..

[31] Sun R., Punyawai P., Furtado N., Afshari F.T., Gallo P. (2025). Paediatric idiopathic syringomyelia—A follow-up of radiological and clinical outcomes into adulthood. Childs Nerv. Syst..

[32] Soo T., Sandquist L., Tong D., Barrett R. (2014). Surgical treatment of idiopathic syringomyelia: Silastic wedge syringosubarachnoid shunting technique. Surg. Neurol. Int..

[33] Fu K.M.G., Smith J.S., Polly D.W., Ames C.P., Berven S.H., Perra J.H., Glassman S.D., McCarthy R.E., Knapp D.R., Shaffrey C.I. (2011). Morbidity and mortality associated with spinal surgery in children: A review of the Scoliosis Research Society morbidity and mortality database: Clinical article. J. Neurosurg. Pediatr..

[34] Pencovich N., Korn A., Constantini S. (2013). Intraoperative neurophysiologic monitoring during syringomyelia surgery: Lessons from a series of 13 patients. Acta Neurochir..

[35] Dlouhy B.J., Dawson J.D., Menezes A.H. (2017). Intradural pathology and pathophysiology associated with Chiari I malformation in children and adults with and without syringomyelia. J. Neurosurg. Pediatr..

[36] Guan J., Yuan C., Zhang C., Ma L., Yao Q., Cheng L., Liu Z., Wang K., Duan W., Wang X. (2020). Intradural Pathology Causing Cerebrospinal Fluid Obstruction in Syringomyelia and Effectiveness of Foramen Magnum and Foramen of Magendie Dredging Treatment. World Neurosurg..

[37] Klekamp J., Batzdorf U., Samii M., Bothe H.W. (1997). Treatment of syringomyelia associated with arachnoid scarring caused by arachnoiditis or trauma. J. Neurosurg..

[38] Gardner W.J. (1965). Hydrodynamic mechanism of syringomyelia: Its relationship to myelocele. J. Neurol. Neurosurg. Psychiatry.

