# Supplementary material for: Idiopathic Syringomyelia: A Systematic Scoping Review
Source: J Clin Med. 2026 Aug 11;15(16):6216. doi: 10.3390/jcm15166216 (PMC13513870; doi:10.3390/jcm15166216)
Supplement: Supplementary file 1 [file jcm-15-06216-s001.zip › jcm-4441871-supplementary-Tables.pdf]

SUPPLEMENTARY MATERIAL

Table S1. Proposed pathogenetic mechanisms

Author, year	Hypothesized mechanism	Supporting evidence
Clifton et al., 1987	Occult intradural arachnoid cyst → CSF block → syrinx	Surgical demonstration of arachnoid cyst; syrinx collapse after fenestration.
Mallucci et al., 1997	Partial subarachnoid block → CSF pressure gradients → syrinx filling	Myelography + intraoperative exploration; collapse after block removal.
Kastrup et al., 2001	Spontaneous resolution due to reorganization of CSF dynamics	8 years serial MRI; complete radiological resolution despite persistent clinical signs.
Bogdanov et al., 2004	Posterior fossa under-development → continuum CM-1 / CM-0 / IS	Morphometric comparison with CM-1 and controls.
Chen et al., 2004	Dural vascular engorgement → cord compression → acute syrinx	Intra-op finding; rapid post-op resolution.
Lin et al., 2006	Disturbed CSF flow at the level of the syrinx	Clinical and radiological improvement after SS shunt.
Nakamura et al., 2009	Two distinct morphological entities: localized vs extended IS, with different CSF dynamics	Morphological stratification + selective myelography.
Struck et al., 2009	Abnormal CSF dynamics at the foramen magnum (Greitz model)	Quantitative cine-MRI: peak systolic caudal velocity +86% vs controls; flow jets and synchronous bidirectional flow in 5/8.
Magge et al., 2011	Pathophysiology heterogeneous and largely unresolved; benign natural history dominant	Long-term clinical and radiological FU in 32 children.
Roy et al., 2011	Convergence on SAS obstruction + intramedullary pulse pressure (Greitz model)	Comprehensive synthesis of all available pathogenetic theories.
Joseph et al., 2013	Disturbed CSF dynamics at multiple levels (cranial and spinal); benign in pediatrics	39 children with stable/improving course; LP responsive in 4/4 with progressive pain.
Soo et al., 2014	CSF flow disturbances without identifiable obstruction	Improvement after silastic-wedge SS shunt.
Rodriguez et al., 2015	Pathogenesis not specifically discussed; emphasis on benign clinical course	Clinical-radiological non-correlation in 98 patients.
Davidson et al., 2017	Subarachnoid CSF block (often subtle) addressable by SS shunting	Surgical exploration; arachnoid pathology found in subset.
Rigsby et al., 2021	IS interpreted as persistent central canal (developmental variant), not a true syrinx	Spontaneous resolution + histopathological reasoning
Lu et al., 2025	Occult arachnoid webs are the substrate of most adult "IS"; entity overlaps with PTDS	100% intraoperative confirmation of webs in 15/15 cases (US, surgical exploration, histology + IHC).
Özkal et al., 2025	Cranial morphometric variants (reduced PF, FM alterations) → continuum CM-0 / IS	Cranial morphometric comparison with controls and CM-1
Sun et al., 2025	Pathophysiology unresolved; hypothesis of misrecognized occult adhesions	Operated case revealed T11 adhesions intra-op.

CM-0, Chiari-zero; CM-1, Chiari malformation type 1; CSF, cerebrospinal fluid; FM, foramen magnum; FU, follow-up; IHC, immunohistochemistry; LP, lumbar puncture; PF, posterior fossa; PSS, primary spinal syringomyelia; PTDS, post-traumatic / post-arachnoiditis dynamic syringomyelia; SAS, subarachnoid space; SM, syringomyelia; SS shunt, syringo-subarachnoid shunt; US, ultrasound.

Table S2. Management strategies and outcomes

Author, year	N. conservative cohort	N. surgical cohort	Surgical technique	Clinical outcomes	Radiological outcomes	Complications	Follow-up
Clifton et al., 1987	0	1	Laminectomy + arachnoid cyst fenestration	Sustained improvement	NA	0	NA
Mallucci et al., 1997	0	10	8 laminectomy + web/pouch/cyst excision; 2 isolated syringo-pleural/peritoneal shunts	5 marked, 3 moderate improvement (laminectomy); 2 deteriorated (shunt-only)	6 collapsed/disappeared, 1 extension, 2 unchanged, 1 deceased before imaging	1 paraplegia post-shunt	NA
Kastrup et al., 2001	1	0	—	Symptoms unchanged	Complete spontaneous resolution	—	8 years
Bogdanov et al., 2004	NA	NA	—	NA	5/17 spontaneous syrinx drainage	—	NA
Chen et al., 2004	0	1	Suboccipital + cervical decompression	Clinical improvement	Syrinx reduction	0	NA
Lin et al., 2006	0	1	Syringo-subarachnoid shunt	JOA 10 → 14	Syrinx reduction	0	1 month
Nakamura et al., 2009	12	3	SS shunt / arachnolysis	Localized IS remained stable; extended IS improved with surgery.	Localized IS (12) remained stable after conservative management; extended IS (3) reduced post-op	—	10 years
Struck et al., 2009	NA	NA	—	NA	NA	—	NA
Magge et al., 2011	30	2	SS shunt / decompression	27/30 (91%) asymptomatic / stable / improved; surgery (2/2) failed to give clinical benefit	87.5% of syringes remained stable, with no correlation with clinical course	0	Clinical 23.8 months; radiological 24.1
Roy et al., 2011	6	2	Decompression / arachnolysis	Conservative cohort: stable; Surgical cohort: improved or stable;	Conservative cohort: 3/6 (50%)stable, no FUP for the others;	0	3 years

					surgical cohort: 2/2 reduced.		
Joseph et al., 2013	35	4	Therapeutic LP (4/4); no permanent shunt	All 39: stable / improved; LP gave significant pain reduction in 4/4	Stable in 35; reduction in 4	0	3.6 years
Soo et al., 2014	0	5	Silastic-wedge SS shunt	5/5 improved	5/5 reduced	0	3–36 months
Rodriguez et al., 2015	97	1	SS shunt	Of the 78 patients with available FUP: 72 (92.3%) improved, 34 (43.6%) remained asymptomatic and stable, 6 (7.7%) deteriorated	Of the 38 patients with available FUP: in 29 (76%) syrinx size remained stable; in 6 (16%) reduced; in 3 (8%) increased	0	Clinical 20.5 months; radiological 13 months
Davidson et al., 2017	0	15	SS shunt	12/15 (80%) improved; 3/15 (20%) stable	On the whole cohort, 37/41 patients (90%) presented radiological reduction	On the whole cohort 3/41 (7%) patients reoperated	36 months
Rigsby et al., 2021	1	0	—	Asymptomatic	Spontaneous resolution	—	3 years
Lu et al., 2025	0	15	Microsurgical excision of arachnoid webs (with intraoperative US)	13/15 (86.7%) improved; 1/15 (6.7%) stable; 1/15 (6.7%) deteriorated	11/15 (73%) significant reduction; 4/15 (26.7%) moderate reduction.	1/15 (6.7%)	24.8 months
Özkal et al., 2025	NA	NA	—	NA	NA	—	Cross-sectional
Sun et al., 2025	27	1	Thoracic arachnoid adhesiolysis	Conservative cohort: 27/27 asymptomatic, stable or improved; 1 operated patient: stable	15/28 (53.6%) significant reduction; 12/28 (42.9%) reduced in height; 1/28 (3.6%) worsened; no association with clinical course	0	Clinical 7.66 years; radiological 6.84 years

JOA, Japanese Orthopaedic Association score; LP, lumbar puncture; NA, not available; POD, post-operative day; PSS, primary spinal syringomyelia; PTDS, post-traumatic / post-arachnoiditis dynamic syringomyelia; SS shunt, syringo-subarachnoid shunt; US, ultrasound.
